# Supplementary material for: Arctic sea-ice loss is projected to lead to more frequent strong El Niño events
Source: Nat Commun. 2022 Aug 23;13:4952. doi: 10.1038/s41467-022-32705-2 (PMC9399112; doi:10.1038/s41467-022-32705-2)
Supplement: Supplementary file 1 — Supplementary Information [file 41467_2022_32705_MOESM1_ESM.pdf]

Supplementary Information for

Arctic sea-ice loss is projected to lead to more frequent strong El Niño events

Jiping Liu, Mirong Song, Zhu Zhu, Radley M. Horton, Yongyun Hu, Shang-Ping Xie

[1]Department of Atmospheric and Environmental Sciences, University at Albany, State University of New York, Albany, NY, USA

[2]State Key Laboratory of Numerical Modeling for Atmospheric Sciences and Geophysical Fluid Dynamics, Institute of Atmospheric Physics, Chinese Academy of Sciences, Beijing, China

[3]Lamont-Doherty Earth Observatory, Columbia University Earth Institute, Palisades, NY, USA

[4]Department of Atmospheric and Oceanic Sciences, School of Physics, Peking University, Beijing, China

[5]Scripps Institution of Oceanography, University of California San Diego, La Jolla, CA, USA

*Corresponding author: Jiping Liu (jliu26@albany.edu)

Supplementary Table 1. Frequency of strong El Niño events in the time slice coupled model experiment with fixed Arctic sea ice during 1980-1999 (ICEhist, row 2) based on the principal component time series associated with the first Empirical Orthogonal Function mode (EOF1_PC). Frequency changes of strong El Niño events in the time slice coupled model experiments with fixed Arctic sea ice during 2020-2039 (ICEp1) and 2080-2099 (ICEp2) relative to that of ICEhist based on EOF1_PC (row 3 and 4, see the caption of Table 1 for the explanation of frequency change).

	EOF1_PC
ICEhist	11.3%
ICEp1−ICEhist	0.3%
ICEp2−ICEhist	**4%**

Bold number means that frequency change is statistically significant (> 95% confidence level) based on the non-parametric bootstrap significant test.

Supplementary Table 2. Frequency changes of strong El Niño events in the time slice Community Climate System Model version 4 (CCSM4) experiment with fixed Arctic sea ice during 2080-2099 (ICEp2_CCSM4) relative to that during 1980-1999 (ICEhist_CCSM4) (row 2) and the time slice Community Earth System Model experiment with fixed sea ice cover only in the North Pacific sector during 2080-2099 (ICEp2NP) relative to that with fixed Arctic sea ice during 1980-1999 (ICEhist) (row 3) (see the caption of Table 1 for the explanation of frequency change).

	ONI(Niño3.4)	Zonal SST gradient	Meridional SST gradient
ICEp2_CCSM4−ICEhist_CCSM4	**8.3%**	**4.3%**	**4.3%**
ICEp2NP−ICEhist	1.7%	0.7%	2%

Bold numbers mean that the frequency changes are statistically significant (> 95% confidence level) based on the non-parametric bootstrap significant test.

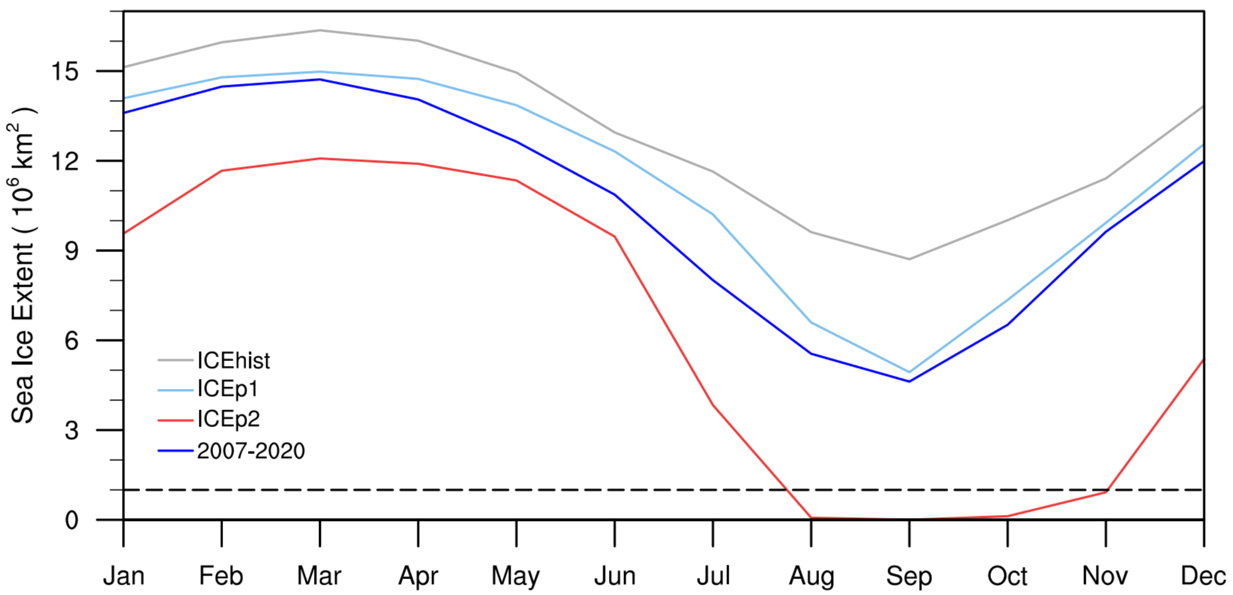

Supplementary Figure 1. The prescribed annual cycle of Arctic sea ice extent in the three coupled model experiments. Gray, light blue, and red lines are our time slice coupled model experiment with fixed Arctic sea ice during 1980-1999 (ICEhist), during 2020-2039 (ICEp1) and 2080-2099 (ICEp2), respectively. The dark blue is the observed ice extent averaged during 2007-2020 (the lowest 14 records since the satellite era). The ice extent below the horizontal dashed line (1 million km^2) is defined as per conventions as "ice-free".

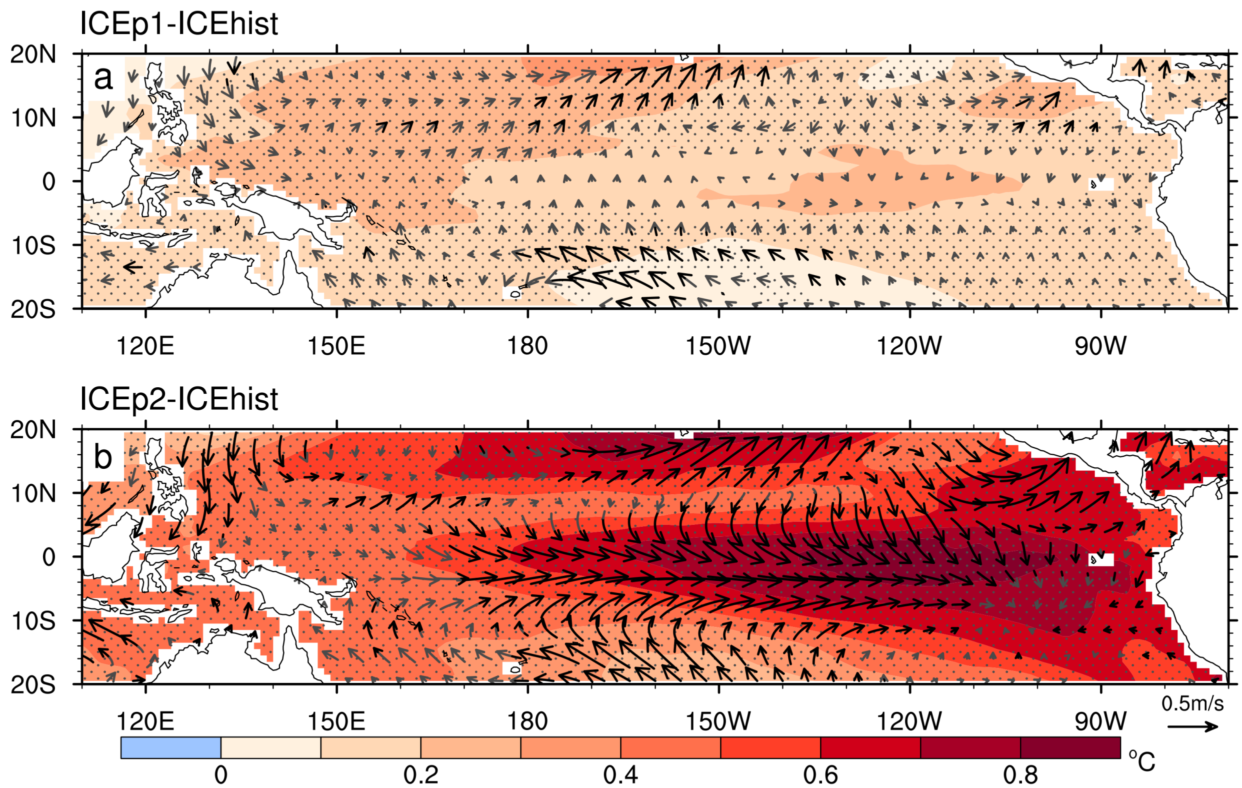

Supplementary Figure 2. Changes in winter sea surface temperature (color shaded, °C) and near surface winds (vector, m s^{-1}) induced by Arctic sea-ice loss. (a) difference between the time slice coupled model experiments with fixed Arctic sea ice during 2020-2039 (ICEp1) and during 1980-1999 (ICEhist), and (b) difference between the time slice model experiments with fixed Arctic sea ice during 2080-2099 (ICEp2) and ICEhist. Statistical significance (> 95% confidence level) is denoted by gray dots and black vectors.

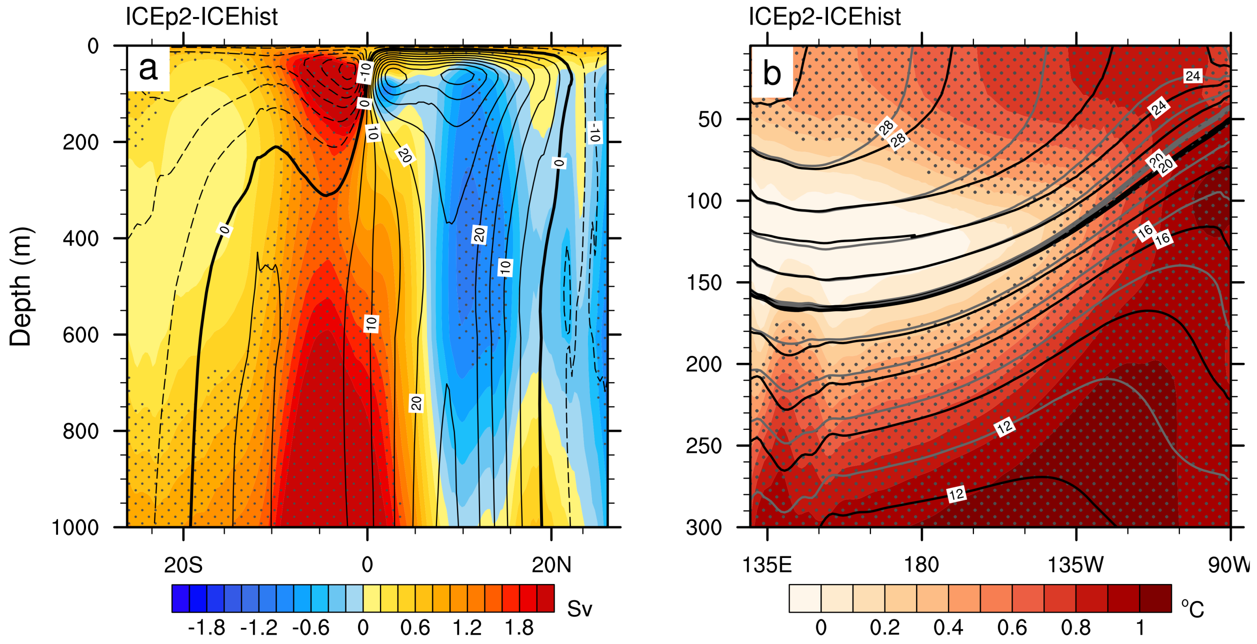

Supplementary Figure 3. (a) Changes in ocean meridional overturning circulation (Sv) zonally averaged across the Pacific (130°E-90°W) between the time slice coupled model experiment with fixed Arctic sea ice during 2080-2099 (ICEp2) and during 1980-1999 (ICEhist). Contours are the climatological stream function of ICEhist. (b) Changes in ocean temperature meridionally averaged across the equatorial Pacific (5°S-5°N, color shaded) between ICEp2 and ICEhist. Contours are the time-mean isotherm (the black line is ICEp2 and the gray line is ICEhist). Statistical significance (> 95% confidence level) is denoted by gray dots.

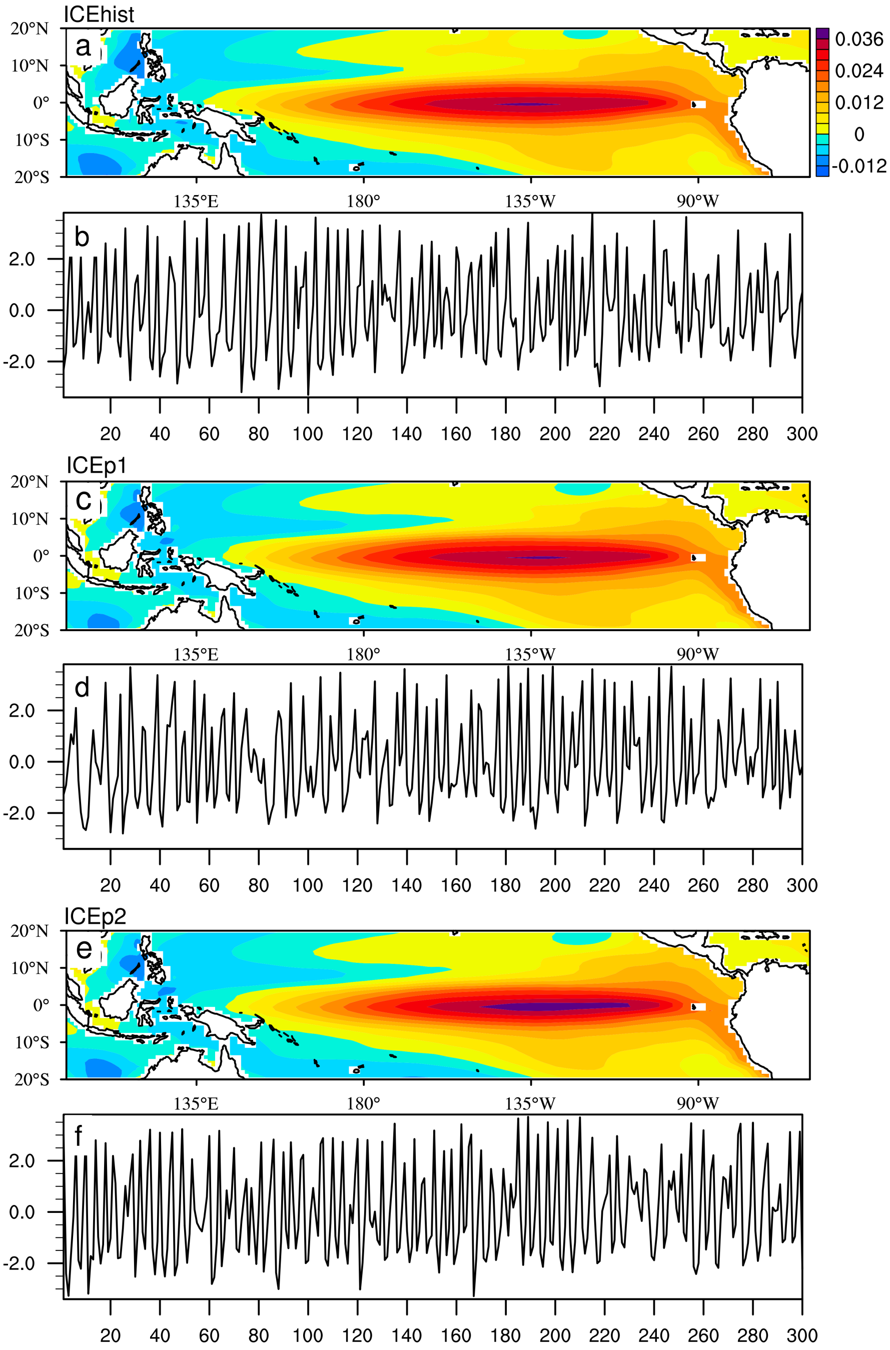

Supplementary Figure 4. The spatial pattern and principal component time-series associated with the first Empirical Orthogonal Function mode of winter sea surface temperature in the tropical Pacific for experiments: (a, b), (c, d), and (e, f) are the time slice coupled model experiment with fixed Arctic sea ice during 1980-1999 (ICEhist), during 2020-2039 (ICEp1) and 2080-2099 (ICEp2), respectively.

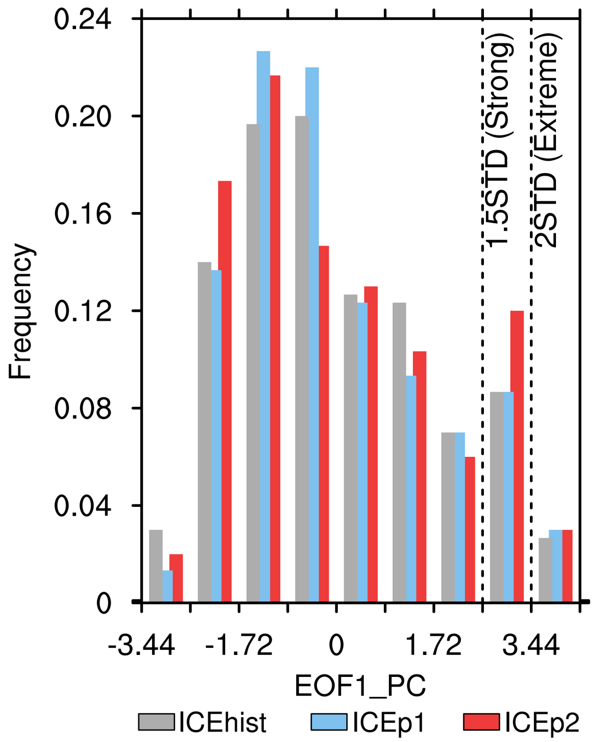

Supplementary Figure 5. Histograms of the ENSO index based on the principal component time-series of the first Empirical Orthogonal Function mode (EOF1_PC). Gray, blue, and red bars are the time slice coupled model experiments with fixed Arctic sea ice during 1980-1999 (ICEhist), during 2020-2039 (ICEp1) and 2080-2099 (ICEp2), respectively. Each bin represents 0.5 standard deviation of the corresponding sea surface temperature anomalies or gradients. Black dashed lines represent 1.5 and 2 standard deviations.

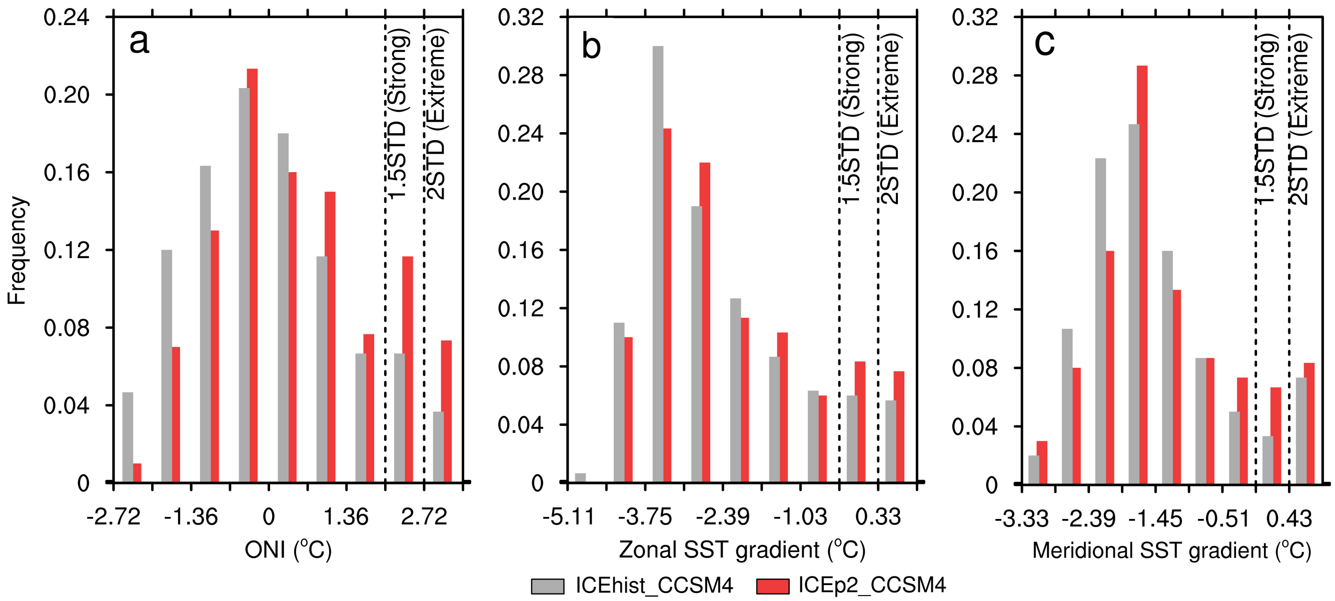

Supplementary Figure 6. Histograms of El Niño indices for the time slice Community Climate System Model version 4 (CCSM4) experiments with fixed Arctic sea ice during 2080-2099 (ICEp2_CCSM4) and during 1980-1999 (ICEhist_CCSM4). (a) the Oceanic Niño Index, (b) the zonal sea surface temperature (SST) gradient, and (c) the meridional SST gradient. Gray and red bars are ICEhist_CCSM4 and ICEp2_CCSM4, respectively. Each bin represents 0.5 standard deviation of the corresponding SST anomalies or gradients. Black dashed lines represent 1.5 and 2 standard deviations.

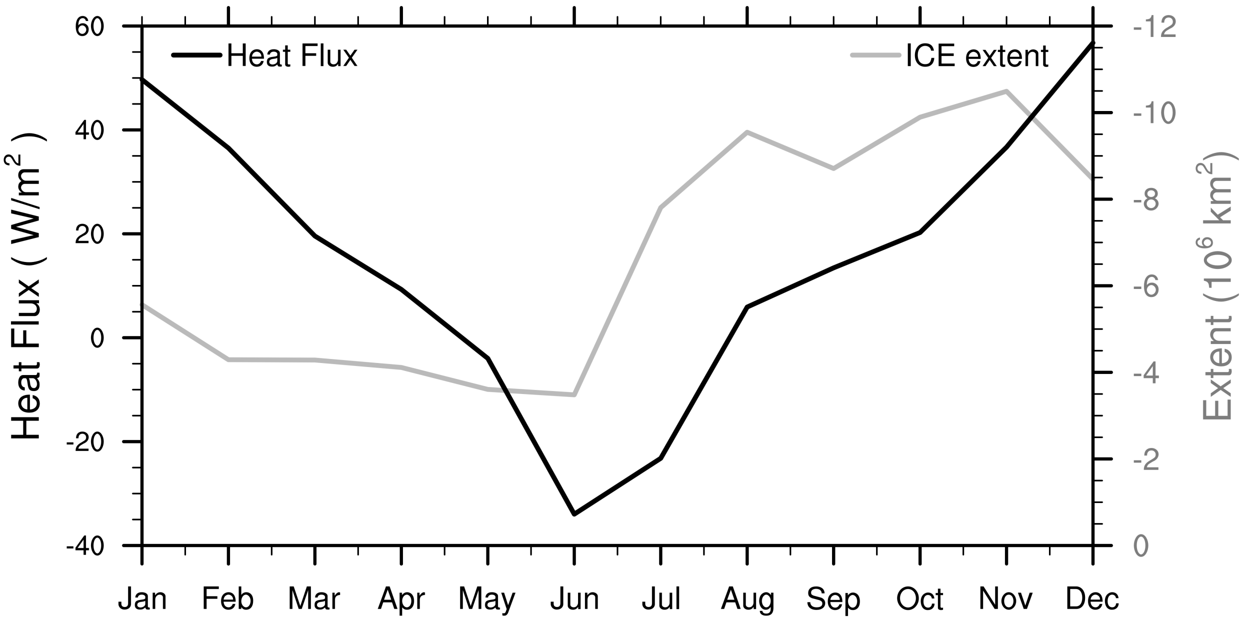

Supplementary Figure 7. Seasonal cycle of changes in the net surface heat fluxes (W m^{-2}, black line) and Arctic sea ice extent (10^6 km^2, gray line) between the time slice coupled model experiments with fixed Arctic sea ice during 2080-2099 (ICEp2) and during 1980-1999 (ICEhist). The net surface heat flux includes the latent and sensible heat fluxes and net shortwave and longwave radiative fluxes.

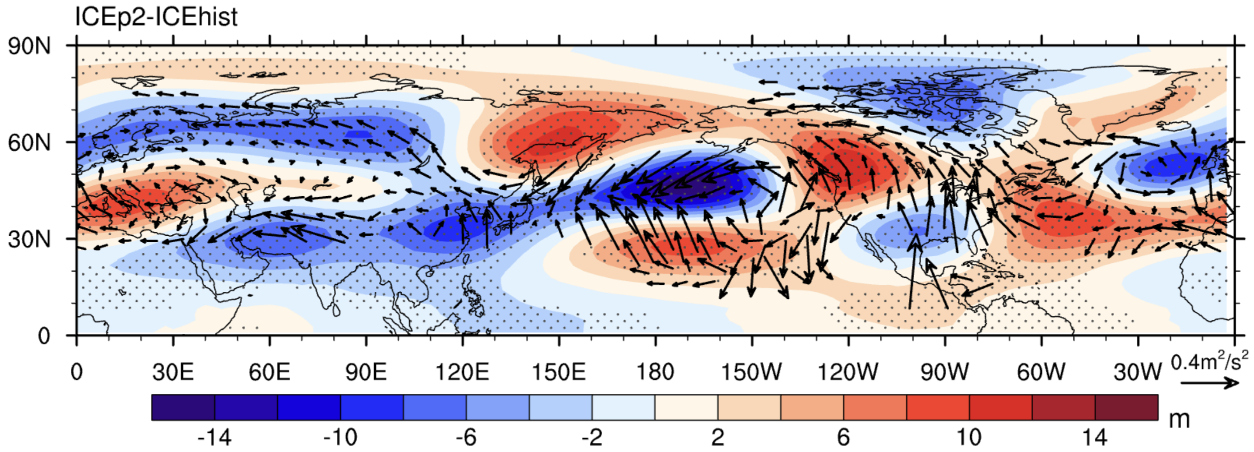

Supplementray Figure 8. Changes in eddy geopotential height (color shaded, m) and wave activity flux (vector, m^2 s^{-2}) at 200-hpa between our time slice coupled model experiment with fixed Arctic sea ice during 2080-2099 (ICEp2) and during 1980-1999 (ICEhist).

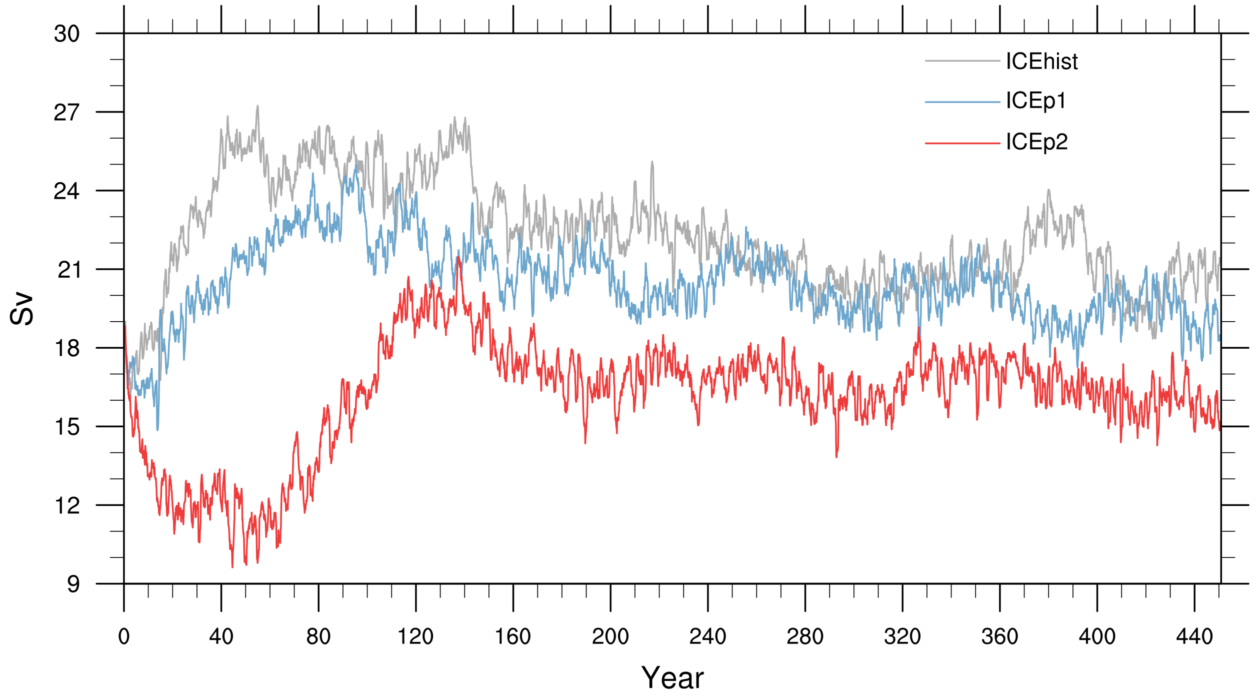

Supplementary Figure 9. Time series of the Atlantic Meridional Overturning Circulation (Sv). Gray, blue, and red lines are our time slice model experiment with fixed Arctic sea ice during 1980-1999 (ICEhist), during 2020-2039 (ICEp1) and 2080-2099 (ICEp2), respectively.

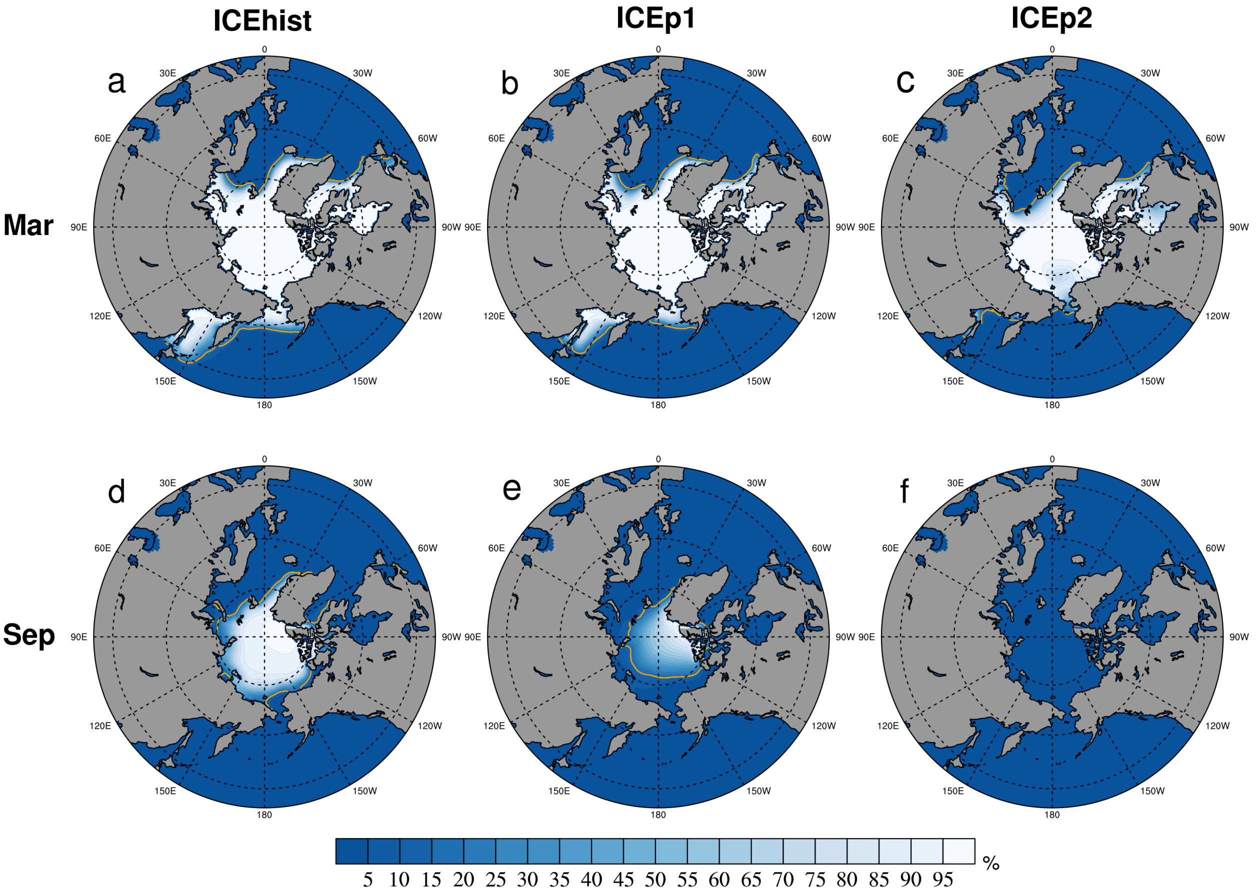

Supplementary Figure 10. The prescribed March (upper panel) and September (lower panel) Arctic sea ice concentrations. (a, d), (b, e), and (c, f) are our time slice model experiment with fixed Arctic sea ice during 1980-1999 (ICEhist), during 2020-2039 (ICEp1) and 2080-2099 (ICEp2), respectively. The orange line represents the sea ice edge defined as the contour of 15% ice concentration.

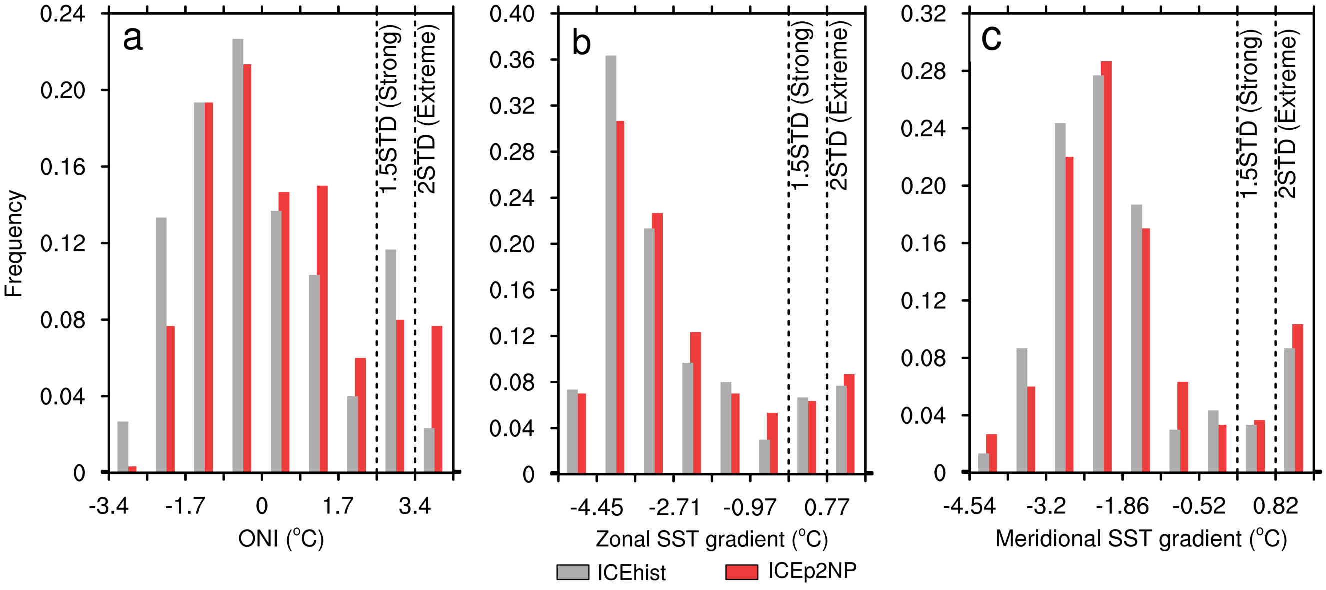

Supplementary Figure 11. Histograms of El Niño indices associated with the time slice coupled model experiment with fixed sea ice cover only in the North Pacific sector during 2080-2099 (ICEp2NP). (a) the Oceanic Niño Index, (b) the zonal sea surface temperature (SST) gradient, and (c) the meridional SST gradient. Red bars are ICEp2NP and gray bars are the time slice coupled model experiment with fixed Arctic sea ice during 1980-1999 (ICEhist). Each bin represents 0.5 standard deviation of the corresponding SST anomalies or gradients. Black dashed lines represent 1.5 and 2 standard deviations.

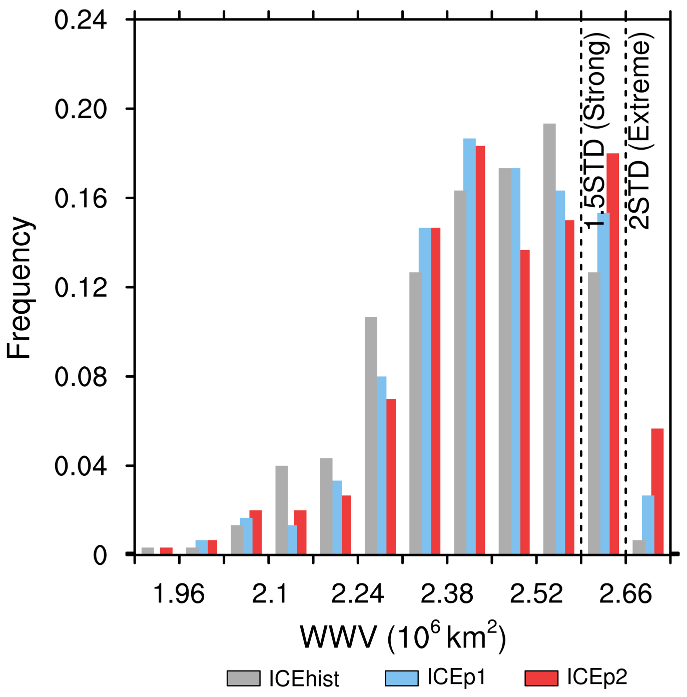

Supplementary Figure 12. Histograms of integrated warm water volume above the 20°C isotherm in the equatorial Pacific associated with Arctic sea-ice loss. Gray, blue, and red bars are our time slice coupled model experiments with fixed Arctic sea ice during 1980-1999 (ICEhist), during 2020-2039 (ICEp1) and 2080-2099 (ICEp2), respectively. Each bin represents 0.5 standard deviations of the integrated warm water volume. The dashed lines represent 1.5 and 2 standard deviations.

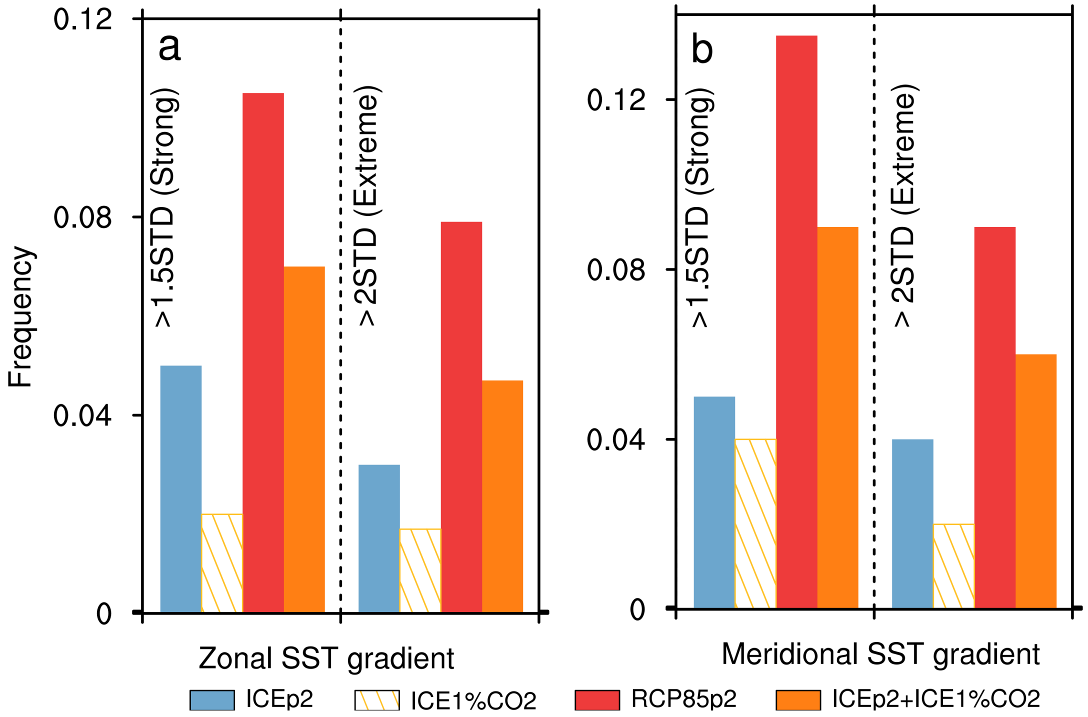

Supplementary Figure 13. The frequency changes of strong (exceeding 1.5 standard deviations) and extremely strong (exceeding 2 standard deviations) El Niño events in the time slice coupled model experiments with fixed Arctic sea ice during 2080-2099 (ICEp2), the 1% per-year CO_2 increase experiment (ICE1%CO_2), and the greenhouse warming experiment during 2080-2099 (RCP85p2) relative to the reference simulation. (a) the zonal sea surface temperature (SST) gradient, and (b) the meridional SST gradient. Blue bars are ICEp2, yellow bars with strips are ICE1%CO_2, red bars are RCP85p2, and orange bars are the combination of ICEp2 and ICE1%CO_2. Bars with stripes mean that the frequency changes are not statistically significant based on the non-parametric bootstrap significant test.
